# A Study on the Application of CO_2_-Modified Atmosphere Combined with Temperature-Control Technology in Rice Warehouse Storage

**DOI:** 10.3390/foods14183217

**Published:** 2025-09-16

**Authors:** Shiming Wang, Yan Zhao, Haoxin Lv, Tianjie Qi, Yongling Song

**Affiliations:** School of Food and Strategic Reserves, Henan University of Technology, Zhengzhou 450001, China; 17637136380@163.com (S.W.); lvhaoxin0129@126.com (H.L.); 15003798360@163.com (T.Q.); yongling80@126.com (Y.S.)

**Keywords:** carbon dioxide-modified atmosphere, temperature control, rice, quality, fungal diversity, AFB1, non-targeted metabolomics, warehouse storage

## Abstract

In recent years, as two globally recognized green grain storage technologies, CO_2_-modified atmosphere (CO_2_) storage and temperature-controlled (TC) storage have gained prominence. However, research on their integrated application remains limited. This study monitored quality dynamics and microbial activity in rice stored for 360 days under CO_2_ + TC versus conventional storage (control), with analyses conducted at stratified sampling points (upper, middle, and lower layers). Compared to conventional storage, CO_2_ + TC preserved rice color more effectively, while retarding the increase in fatty acid value and the decline in brown rice yield, head rice yield, and germination percentage. Furthermore, CO_2_ + TC storage effectively suppresses the proliferation of *Fusarium* and *Aspergillus*, thereby retarding aflatoxin B1 (AFB1) accumulation by inhibiting fungal metabolic activity. Non-targeted metabolomics analysis further verified that CO_2_ + TC storage enhanced rice antioxidant capacity and disease resistance by modulating amino acid, carbohydrate, and linolipid metabolic pathways. This technology effectively maintained nutrient retention (e.g., amino acids and proteins) and delayed quality deterioration in stored rice. These findings elucidated the underlying mechanism of CO_2_ + TC on rice quality, offering a novel perspective for grain storage technology.

## 1. Introduction

Rice (*Oryza sativa*) constitutes one of the primary cereal crops globally [1], sustaining approximately half of the world’s population as a staple food source. Asia dominates global rice production, contributing over 90% of the world’s output [2]. Approximately 60% of China’s population relies on rice and its processed products as staple food. Due to the seasonal nature of rice production and the continuity of consumption, it is necessary to store newly harvested rice for a period of time to prevent crop failure or poor yields in the following year. However, during conventional warehouse storage, paddy rice is highly susceptible to elevated temperatures and mycotoxin-producing molds, leading to accelerated physicochemical deterioration and complete loss of seed viability, resulting in significant losses [3]. Therefore, identifying optimal storage conditions for rice in actual warehouse environments is critical to retard quality degradation during warehouse storage [4].

The quality deterioration of rice during warehouse storage results from the coupled effects of multiple factors, including temperature, humidity, gas composition, biotic stressors, and storage duration [5]. To mitigate such deterioration, prevailing approaches integrate chemical fumigation, biological control agents, modified atmosphere technology, and temperature-regulated systems [6]. CO_2_-modified atmosphere technology, as a green storage method, demonstrates significant efficacy in grain preservation [7]. Studies confirm that elevated CO_2_ concentrations (>35%) effectively suppress insect infestations and inhibit grain respiration alongside microbial proliferation [8]. For example, Gupta et al. (2014) [9] reported that high CO_2_ levels restrict microbial growth on rice surfaces, thereby delaying quality deterioration and extending storage longevity. Similarly, Sun et al. (2019) [10] found that CO_2_-treated rice exhibited markedly reduced changes in fatty acid value, cooked rice hardness, lipoxygenase activity, and α-amylase activity compared to conventional storage. Collectively, these effects retard quality degradation, prolong storage duration, and enhance economic returns [11]. As a green grain storage technology, CO_2_-modified atmosphere storage is characterized by rapid insecticidal action and low concentration requirements. It effectively replaces traditional chemical fumigation methods like hydrogen phosphide, thereby eliminating chemical residues, preventing pest resistance development, and mitigating environmental pollution [12].

Temperature represents a critical factor in rice storage. Temperature-controlled grain storage technology, now highly mature, enhances storage quality, prolongs shelf life, and reduces losses through precise regulation of thermal conditions within storage environments. As a green, efficient, and safe preservation method, it has been extensively researched. For example, Han et al. (2024) [13] elucidated that elevated temperatures accelerate lipid oxidation and increase free fatty acid content, exacerbating quality degradation. Qu et al. (2023) [14] established that hypothermal preservation (≤15 °C) significantly retards color alteration in elevated-moisture rice. Further supporting this, Abdelfattah et al. (2020) [15] revealed divergent microbial community structures and grain quality outcomes under varying storage temperatures, with quasi-low temperature conditions (16–20 °C) effectively suppressing microbial proliferation. Collectively, these findings indicate that low-temperature storage optimizes grain preservation by inhibiting spoilage microorganisms and mitigating biochemical deterioration.

Non-targeted metabolomics (NTM) is a hypothesis-generating, global, and unbiased analytical method for all small-molecule metabolites in a biological system under given conditions [16]. NTM can detect a variety of metabolites, providing a comprehensive solution for the metabolite profiling of biological samples [17]. It has now been widely applied in numerous fields such as medicine, agronomy, and grain storage. For example, Chen et al. (2022) [18] identified potential biomarkers associated with the occurrence of grade I ischemic apoplexy using untargeted metabolomics approaches. Xu et al. (2020) [19] delineated cold-adaptation mechanisms in tobacco through integrated transcriptomic–metabolomic analysis, revealing metabolic pathway rearrangements. Li et al. (2024) [20] deciphered peanut responses to hypoxic re-aeration storage via combined proteomic–metabolomic profiling. However, no study has yet applied non-targeted metabolomics (NTM) to investigate metabolite dynamics in warehouse-stored rice under CO_2_ + TC. Therefore, it is necessary to systematically study the effects of CO_2_-modified atmosphere combined with temperature-controlled technology on the quality deterioration of rice, its mechanisms in storage and processing quality, as well as the involved metabolic regulation pathways. This study employed the UHPLC-Orbitrap-Exploris-MS (UHPLC-OE-MS)-based NTM method to analyze differentially expressed primary metabolites in rice grains treated with CO_2_-modified atmosphere combined with temperature-controlled technology, and conducted a comparative analysis of primary metabolite differences among different treatments.

Although previous studies have examined the individual effects of temperature control or CO_2_-modified atmosphere on rice quality and metabolites during storage, research on integrated CO_2_-modified atmosphere with temperature-control technology in actual warehouse settings remains limited. To address this gap, this study aimed to assess the storage stability of rice under combined CO_2_-modified atmosphere and temperature-control conditions by evaluating key quality indicators—including color, fatty acid value, brown rice rate, head rice yield, germination percentage—alongside microbial diversity and aflatoxin B1 content. NTM was further employed to analyze rice samples across storage conditions. Metabolomic pathway mapping based on differential metabolites elucidated the underlying mechanisms, thereby providing a scientific foundation for scaling up this integrated technology in warehouse rice storage.

## 2. Materials and Methods

### 2.1. Materials

Two large warehouses with identical specifications (length: 42 m, width: 24 m, grain stacking height: 6 m, designed rice storage capacity: 3300 t) were selected from a grain depot in Anhui Province (30.96 °N, 117.82 °E).

Experimental warehouse: Integrated winter mechanical ventilation and summer HVAC-assisted temperature control maintained the average grain temperature at ≤15 °C year-round, with local maxima ≤25 °C. CO_2_-modified atmosphere fumigation (CO_2_ ≥ 35% *v*/*v*) was applied during grain intake and late July of the subsequent year. During CO_2_ treatment, CO_2_ concentration within the grain warehouse was sustained at ≥35% for a minimum of 15 consecutive days. Control warehouse: Only conventional storage practices—including mechanical ventilation and chemical fumigation—were implemented, without HVAC-assisted temperature control or CO_2_-modified atmosphere technology. The humidity level in both storage bins was controlled within the range of 55% to 65% throughout the experiment.

The stored rice was early indica rice produced in 2023 in Anhui Province, China, with a moisture content of 13.4%, stored from late July 2023. Samples were collected from geometric centers of experimental and control warehouses (central surface point and mid-depth at 50% grain stack height) after grain leveling. These samples were pooled to form origin samples. This sampling timepoint was designated as the experiment commencement. Then, triplicate samples were collected from the upper, middle, and lower strata at the geometric center of the grain bulk at 90-day intervals. Sampling depths were defined as follows: upper stratum (0.5 m below grain surface), middle stratum (50% grain stack height), and lower stratum (0.5 m above floor level). Total storage duration was 360 days.

### 2.2. Rice Color Measurement

Colorimetric parameters were measured according to Wu et al. (2023) [21]. Surface color parameters of high-moisture Japanese brown rice were measured using an NR200 colorimeter (3NH Technology Co., Ltd. Shenzhen, China). Following a 15-min warm-up, the instrument was calibrated with a standard white reference plate. Untreated samples served as controls. The measured chromaticity parameters comprised L* (lightness, higher values indicate greater brightness), a* (red–green axis, positive values signify red intensity, negative values signify green intensity), and b* (yellow–blue axis, positive values signify yellow intensity, negative values signify blue intensity).

### 2.3. Fatty Acid Value (FAV)

FAV was determined according to Zhang’s method (2009) [22] with modifications: Rice samples were dehulled using an impact sheller (Model BLH-3250B; Bethlehem Instruments Ltd., London, UK). Dehulled grains were ground to a fine powder (≤0.5 mm) using a cyclone mill (Model TDW-5000; Tongxin Tianbo Technology, Beijing, China). The powder was mixed with absolute ethanol (HPLC grade), vigorously vortexed for 2 min, and filtered through Whatman No.1 paper. The filtrate was combined with CO_2_-free distilled water (blank: ethanol-substituted filtrate) and titrated with 0.01 M KOH standard solution using phenolphthalein indicator (4 drops). FAVs were calculated as the volume of KOH required to neutralize free fatty acids per 100 g rice sample, expressed as milligrams KOH per 100 g dry matter (mg KOH/100 g dm).

### 2.4. Brown Rice Yield (BRY)

BRY was measured according to the methods of Dou et al. (2024) [23] with modifications: A sample of 20–25 g (*m*_0_) was taken from cleaned rough rice. After dehulling, the brown rice mass was weighed (*m*_1_). The remaining grains were mechanically husked, and the total brown rice mass was recorded (*m*_2_). Defective kernels were sorted from brown rice and weighed (*m*_3_). The units of *m*_0_, *m*_1_, and *m*_2_ are g. BRY (%) was calculated as (1):

(1) $$X\;=\;\frac{\left(m_{1}\;+\;m_{2}\right)\;-\;(m_{1}\;+\;m_{3})/2}{m_{0}}\;\times \;100$$

### 2.5. Head Rice Yield (HRY)

HRY was measured according to Shad’s method (2020) [24] with modifications: 20–25 g sound paddy (*m*_0_) was dehulled using a calibrated laboratory husker (THU35C-C, Satake Corporation, Riichi Satake, Hiroshima, Japan). Unhusked grains were manually separated from brown rice and reprocessed until complete hulling. The resultant brown rice was milled in a standardized milling machine (JGMJ8098, Jiading Grain and Oil Instrument Co., Ltd., Shanghai, China). Milling duration was optimized (120 ± 5 s) to achieve Grade 3 rice precision. Bran powder was removed by aspiration, and intact head rice kernels were sorted and weighed (*m*). The units of *m*_0_ and *m* are g.

### 2.6. Germination Percentage

Germination percentage was measured according to the methods of Chao et al. (2021) [25] with modifications: Glass Petri dishes (90 mm, REBIO, Shanghai, China) and tools were autoclaved (121 °C, 20 min). Sterile quartz sand (particle size 0.5–1.0 mm, Shanghai Baishengyue Biotechnology Co., Ltd., Shanghai, China) was layered over 3 sheets of filter paper in Petri dishes, moistened with sterile water to 80% WHC (water holding capacity). A total of 100 sound grains were aseptically arranged at 5 mm intervals on the substrate. Samples were maintained at 30 ± 0.5 °C with 90% RH in darkness for 7 d, with daily replenishment of sterile water to maintain constant moisture.

### 2.7. Fungal Diversity

Sample collection and preparation: Fungal diversity analysis was conducted exclusively on origin samples (t = 0) and post-storage specimens (360 d). Sampling details are provided in Table 1. The total genomic DNA of fungal communities was extracted using the E.Z.N.A.^®^ Soil DNA Kit (Omega Bio-tek, Norcross, GA, USA), with DNA quality assessed by 1% agarose gel electrophoresis. The V3-V4 hypervariable region of fungal ITS genes was amplified via PCR with barcoded primers 338F (5′-ACTCCTACGGGAGGCAGCAG-3′) and 806R (5′-GGACTACHVGGGTWTCTAAT-3′). Paired-end sequencing (2 × 250 bp) was conducted on the Illumina (San Diego, Ca, USA) NextSeq 2000 platform.

Bioinformatic analysis of rice-associated fungi: Raw sequencing reads were assembled and quality-filtered. Quality-controlled sequences were clustered into operational taxonomic units (OTUs) at a 97% similarity threshold using USEARCH v11, followed by chimera removal with the UCHIME algorithm.

### 2.8. Determination of Aflatoxin B1 (AFB1) Content

AFB1 in rice was determined according to the methods of Alwan et al. (2022) [26] with modifications: This study utilized the RIDASCREEN^®^ AFB1 30/15 detection kit (R-Biopharm, Darmstadt, Germany; Cat. No. R1211). The kit protocol was followed.

### 2.9. Metabolomics Analysis

Following 360 days of storage, tri-level sampling (upper/mid/lower strata) was performed at the geometric centroids of the experimental and control warehouses (*n* = 6 locations). With the inclusion of origin specimens (t = 0), seven independent sample sets were constituted. Each set underwent quadruplicate preparation for subsequent analyses.

#### 2.9.1. Sample Preparation

A total of 50 mg rice grains were transferred to a 2 mL microcentrifuge tube containing one 6 mm stainless steel grinding bead. A total of 400 μL of ice-cold extraction solvent (methanol/water = 4:1, *v*/*v*) spiked with 0.02 mg/mL internal standard (L-2-chlorophenylalanine) was added. Cryogenic homogenization was performed at −10 °C and 50 Hz for 6 min using a ball mill (e.g., MM 400, Retsch, Haan, Germany). Ultrasonic-assisted extraction followed at 5 °C and 40 kHz for 30 min (e.g., Sonorex Digitec DT 255). Samples were incubated at −20 °C for 30 min, then centrifuged at 13,000× *g* (4 °C, 15 min). The supernatant was transferred to LC-MS vials equipped with low-volume inserts (e.g., 250 μL, glass).

#### 2.9.2. Quality Control (QC) Sample

QC samples were prepared by pooling equal volumes of metabolites from all experimental samples. During instrumental analysis, one QC sample was inserted per every 5–15 test samples to monitor the reproducibility of the analytical workflow.

#### 2.9.3. LC-MS/MS Analysis

Samples were analyzed using ultra-high performance liquid chromatography coupled to Fourier transform mass spectrometry (UHPLC-QExactive HF-X system; Thermo Fisher Scientific, Waltham, MA, USA) via a local service provider (Meiji Biomedical Technology Co., Ltd., Shanghai, China).

Chromatographic conditions: 3 μL samples were separated on a Waters HSS T3 column (100 mm × 2.1 mm i.d., 1.8 μm) and detected by MS. Mobile phase A: H_2_O/ACN (95: 5, *v*/*v*) + 0.1% formic acid. Mobile phase B: ACN/IPA/H_2_O (47.5:47.5:5, *v*/*v*/*v*) + 0.1% formic acid. Flow rate: 0.40 mL/min. Column temperature: 40 °C.

Mass spectrometric conditions: The sample mass scan mode was positive/negative ion switching, and the mass scanning range was *m/z* 70-1050. The sheath gas pressure was 50 psi, the auxiliary gas pressure was 13 psi, the auxiliary gas temperature was 425 °C, the positive mode ion spray voltage was set to +3.5 kV, the negative mode ion spray voltage was set to −3.5 kV, the capillary temperature was 325 °C, and the normalized collision energy (NCE) was stepped at 20%, 40%, and 60%. The resolution of the primary mass spectrum was 60,000 FWHM, and the resolution of the secondary mass spectrum was 7500 FWHM, and the acquisition mode was data-dependent acquisition (DDA).

#### 2.9.4. Metabolomic Data Processing

LC-MS raw data preprocessing was conducted using the Progenesis QI software (v2.3; Waters Corporation, Milford, MA, USA). Metabolite identification was performed via the Human Metabolome Database (HMDB; www.hmdb.ca). Multivariate statistical analyses, including principal component analysis (PCA) and orthogonal partial least squares–discriminant analysis (OPLS-DA), were implemented with the R package ropls (v1.6.2). Hierarchical clustering analysis (HCA) and metabolic pathway analysis were executed using the Python library SciPy (v1.0.0). Metabolic pathway annotation was achieved by mapping identified metabolites to the KEGG PATHWAY database (www.kegg.jp/kegg/pathway.html (accessed on 16 June 2025)).

### 2.10. Data Processing

All experiments were independently repeated three times. Data were recorded and preliminarily analyzed using Excel. Statistical analysis was performed using one-way analysis of variance (ANOVA) in SPSS 16.0, and graphical representations were generated using Origin 2021. Significant differences (*p* < 0.05) were determined by Duncan’s multiple range test. Results are presented as mean ± standard deviation (SD).

## 3. Results and Discussion

### 3.1. The Origin Rice Quality Parameters

Prior to storage, the original rice samples were analyzed for color and fatty acid value. Additionally, rough rice yield, head rice yield, germination percentage, and aflatoxin B1 content were quantified. Results are presented in Table 2.

### 3.2. The Color Changes in Rice During Storage

**Figure 1:**
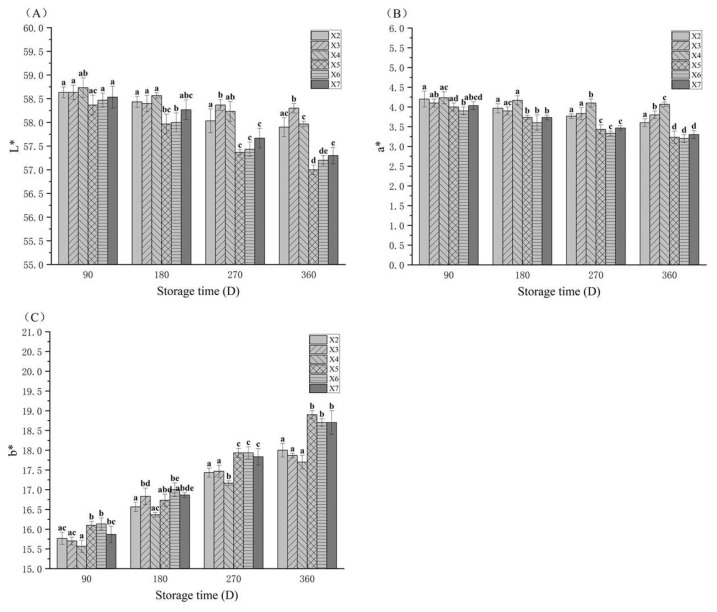
Changes in the L* (**A**), a* (**B**), and b* (**C**) of rice samples that were stored under various conditions. X2, X3, and X4 correspond to the top, middle, and bottom rice samples of the experimental warehouse; X5, X6, and X7 correspond to the top, middle, and bottom rice samples of the control warehouse. In the graph, different letters indicate significant differences at the same storage duration (*p* < 0.05).

The deterioration in stored rice quality was also reflected in the husk color. As shown in Figure 1, with increasing storage duration, the L* and a* values of rice stored in all layers of both the experimental and control silos decreased, while the b* value increased. This trend aligns with the findings reported by Wang et al. (2022) [27]. From 270 to 360 days of storage, the L* value of rice stored in the experimental warehouse was significantly higher than that in the control warehouse in the corresponding layer during the same period (*p* < 0.05). From 180 to 360 days of storage, the a* value of rice stored in the experimental warehouse was significantly higher than that in the control warehouse in the corresponding layer during the same period (*p* < 0.05). From 270 to 360 days of storage, the b* value of rice stored in the experimental warehouse was significantly lower than that in the control warehouse in the corresponding layer during the same period (*p* < 0.05). Combined with the origin data in Table 2, the experimental warehouse demonstrated significantly lower variation in L*, a*, and b* values of stored rice compared to the control warehouse (*p* < 0.05). In conclusion, integrated controlled atmosphere (CA) with CO_2_ enrichment and temperature control effectively delayed the decline in rice grain brightness (L*) and redness (a*), and inhibited excessive yellowness (b*) development. Similarly, Kibal et al. (2021) [28] documented that low-temperature storage delayed pericarp color darkening in quinoa grains. Additionally, Gao et al. (2021) [29] demonstrated that CO_2_-enriched controlled atmosphere storage effectively decelerated appearance deterioration in rice kernels. These findings collectively align with the experimental results of the current research.

### 3.3. The Fatty Acid Value (FAV) Changes in Rice During Storage

**Figure 2:**
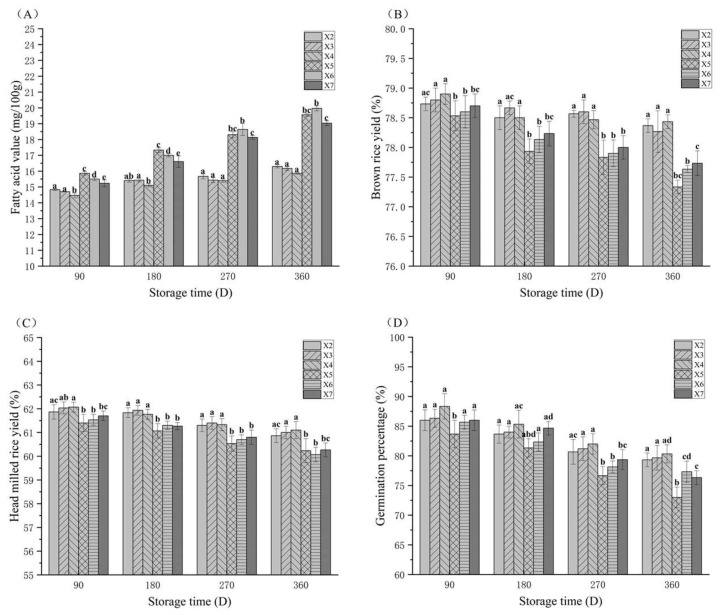
Changes in the FAV (**A**), brown rice yield (**B**), head milled rice yield (**C**), and germination percentage (**D**) of rice samples that were stored under various conditions. X2, X3, and X4 correspond to the top, middle, and bottom rice samples of the experimental warehouse; X5, X6, and X7 correspond to the top, middle, and bottom rice samples of the control warehouse. In the graph, different letters indicate significant differences at the same storage duration (*p* < 0.05).

FAV, defined as the content of free fatty acids released from lipids, serves as a key indicator to quantify the extent of lipid hydrolysis in rice grains during storage. Excessive accumulation of these free fatty acids induces rancidity in rice, significantly compromising its edible quality and sensory properties [30]. As shown in Figure 2A, the FAV of rice in both the experimental and control warehouses demonstrated a consistent upward trajectory throughout the storage period, aligning closely with the findings reported by Zhao et al. (2021) [2]. Throughout the storage period from 90 to 360 days, the FAV of rice in the experimental warehouse remained consistently and significantly lower than that in the control warehouse at equivalent sampling depths (*p* < 0.05). Integrated with the origin data in Table 2, the incremental rise in FAV of rice stored in the experimental warehouse was significantly attenuated compared to the control warehouse throughout the storage period. These findings demonstrate that the synergistic application of CO_2_-controlled atmosphere and precision temperature control effectively retards FAV elevation in stored rice. Similarly, Wang et al. (2021) [31] demonstrated that prolonged storage duration induced a significant increase in the FAV of rice across all temperature regimes, whereas lower temperatures attenuated the FAV growth rate. Sun et al. (2019) [10] observed that rice under CO_2_-controlled atmosphere exhibited a reduced incremental rise in FAV compared to non-controlled storage, indicating that CO_2_-controlled atmosphere retards FAV elevation and mitigates rice deterioration. These findings align with our experimental data.

### 3.4. The Brown Rice Yield (BRY) Changes in Rice During Storage

BRY is a grading indicator for rice grains, directly reflecting their processing quality. As shown in Figure 2B, the brown rice rate in both experimental and control warehouses progressively declined throughout storage, aligning with Tian et al. (2025) [32]. Throughout the 90–360-day storage period, BRY in the experimental warehouse remained significantly higher than that in the conventional control warehouse (*p* < 0.05). Integrated with Table 2 origin data, the attenuation of BRY decline in the experimental warehouse was lower than that observed in the control group over the storage period. These findings demonstrate that integrated CO_2_-modified atmosphere and temperature control effectively retard the degradation of BRY by suppressing lipase activity and microbial proliferation, thereby preserving kernel integrity during storage. Similarly, Ziegler et al. (2024) [33] demonstrated that brown rice rate is significantly influenced by storage temperature, with lower temperatures attenuating the decline in BRY. These findings align with our experimental data.

### 3.5. The Head Rice Yield (HRY) Changes in Rice During Storage

HRY, defined as the mass percentage of milled rice kernels retaining at least three-quarters of their original length, is a critical indicator of rice processing quality and directly determines its market value. As shown in Figure 2C, HRY progressively declined in both experimental and control warehouses throughout storage. This attenuation aligns with Wang et al. (2017) [34], who attributed HRY loss to grain fissure propagation and microbial degradation of the starch–protein matrix. During the 180–360-day storage period, the HRY in the experimental warehouse remained significantly higher than that in the conventional control warehouse within the same storage layer (*p* < 0.05). Integrated with Table 2 origin data, the attenuation of HRY decline in the experimental warehouse was lower than that observed in the control group over the storage period. These findings demonstrate that integrated CO_2_-modified atmosphere and temperature-control technology effectively retards the decline rate of HRY. Similarly, Liu et al. [35] reported that CO_2_-enriched environments attenuate the decline in HRY. These findings align with our experimental data.

### 3.6. The Germination Percentage Changes in Rice During Storage

As shown in Figure 2D, the germination percentage of rice stored in both experimental and control warehouses exhibited a declining trend with prolonged storage duration, consistent with the findings reported by Hu et al. (2022) [36]. During the 270–360-day storage period, the germination percentage of rice in the experimental warehouse was significantly higher than that in the control warehouse at identical storage durations and vertical layers (*p* < 0.05). Comparative analysis with origin data (Table 2) confirmed a marked reduction in germination rate decline within the experimental warehouse. This demonstrates that integrated CO_2_-modified atmosphere and temperature-control technology effectively delays the deterioration of seed vitality and maintains superior physiological activity in stored rice. Similarly, Aguiar et al. (2015) [37] demonstrated that elevated carbon dioxide concentrations preserve seed germination capacity and viability while suppressing fungi associated with seeds, such as Aspergillus and Fusarium, findings consistent with our experimental results.

### 3.7. The Diversity of Fungi Changes in Rice During Storage

**Figure 3:**
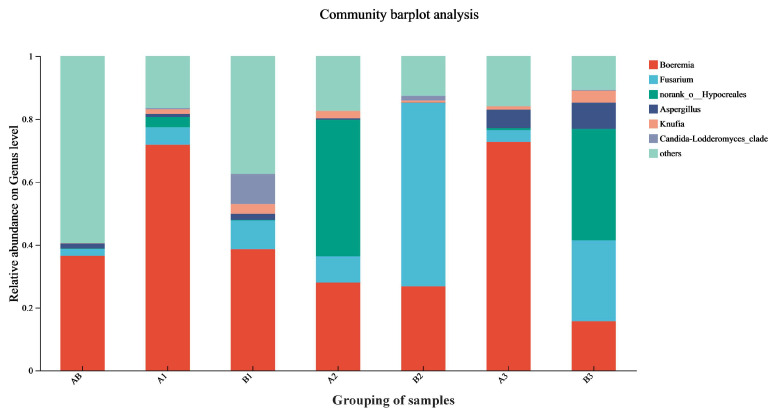
Comparison of the relative abundance of genus fungal species in samples from each layer of the rice experimental and control warehouses (the horizontal axis represents the name of the sample, A and B represent the experimental and control warehouses, the numbers 1, 2, and 3 represent the upper, middle, and lower layers, while AB represents the origin rice).

Figure 3 shows that *Boeremia* was the dominant genus at origin, representing 36.56% of the microbial community. After 360 days of storage, *Boeremia* became dominant in the upper (71.82%) and lower layers (72.75%) of the experimental warehouse, as well as in the upper layer (38.62%) of the control warehouse. Distinct fungal succession occurred in the middle layer of the experimental warehouse, the middle layer of the control warehouse, and the lower layer of the control warehouse. In the middle layer of the experimental warehouse, *norank-o-Hypocreales* increased to 43.31% relative abundance. In the middle layer of the control warehouse, *Fusarium* fungi surged to 58.33% relative abundance. In the lower layer of the control warehouse, *Fusarium* and *norank-o-Hypocreales* fungi rose to 25.72% and 35.45% relative abundance, respectively. These findings are consistent with the results reported by Hashem et al. (2021) [38]. *Boeremia* remained the dominant genus in all other rice samples.

Experimental results demonstrated distinct fungal succession in rice stored across both warehouses after 360 days. *Boeremia* dominated the baseline samples with a relative abundance of 36.56%. After storage, its abundance varied across layers: in the experimental warehouse, values reached 71.82% (upper), 38.62% (middle), and 28.01% (lower); in the control warehouse, levels were 26.71% (upper), 72.75% (middle), and 15.71% (lower). However, although *Boeremia* dominated as the predominant fungal genus in pre-storage rice, no empirical evidence indicates its impact on storage processes, consistent with its classification as a field fungus [39]. Initially, *Fusarium* accounted for 2.12% of the fungal community. After 360 days of storage, its relative abundance in the control warehouses increased significantly to 58.33% (middle layer) and 25.72% (lower layer), indicating pronounced fungal succession. Similarly, *Aspergillus* increased from an initial 1.59% to 1.67% (upper, experimental warehouses), 1.91% (lower, experimental warehouses), 5.84% (upper, control warehouses), and 8.34% (lower, control warehouses), demonstrating progressive colonization across strata, which was similar to the results of Femenias et al. (2024) [40]. Compared with conventional storage, integrated CO_2_-modified atmosphere and temperature-control technology effectively suppresses the proliferation of *Fusarium* and *Aspergillus* fungi during grain storage. This finding aligns with the research by Kushwaha et al. (2023) [41], which demonstrated that combining low temperature (20 °C) with a carbon dioxide-modified atmosphere (35% *v*/*v*) effectively suppressed the growth of *Fusarium* and *Aspergillus* during storage. *Fusarium* species produce mycotoxins such as *Aflatoxins*, *Fumonisins*, and *Deoxynivalenol* during storage [42]. Elevated relative abundance of *Aspergillus* fungi may lead to *Aflatoxin* and *Ochratoxin* production [43], posing significant threats to global food safety. Therefore, it is speculated that the implementation of CO_2_-modified atmosphere and temperature-control technology will affect the content of mycotoxins in rice during storage.

### 3.8. The AFB1 in Rice During Storage

**Figure 4:**
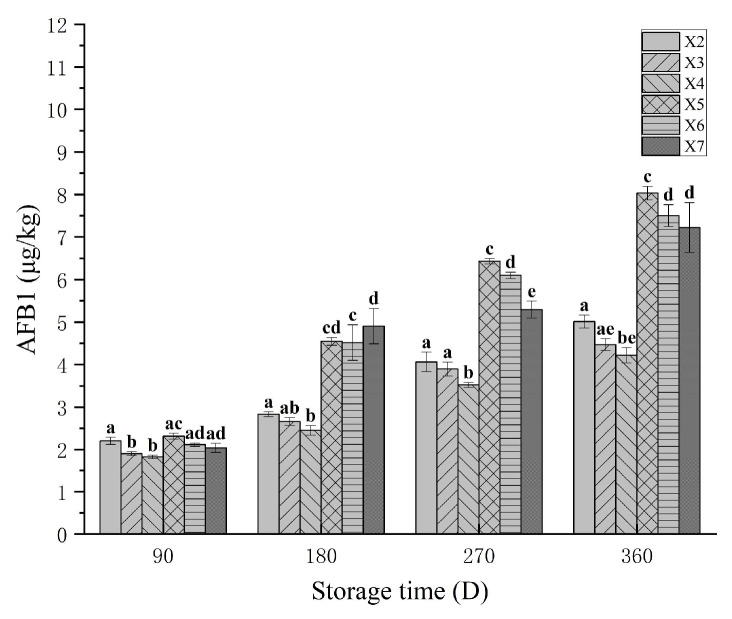
Changes in the AFB1 content of rice samples that were stored under various conditions. X2, X3, and X4 correspond to the top, middle, and bottom rice samples of the experimental warehouse; X5, X6, and X7 correspond to the top, middle, and bottom rice samples of the control warehouse. In the graph, different letters indicated significant differences at the same storage duration (*p* < 0.05).

Aflatoxin B1 (AFB1) serves as a critical indicator of rice quality and safety, directly determining the food safety of rice products. As shown in Figure 4, AFB1 levels in rice increased progressively across the upper, middle, and lower layers of both experimental and control bins during storage. This trend aligns with findings by Ouma et al. (2024) [44], confirming time-dependent AFB1 accumulation under typical storage conditions. From 180 to 360 days of storage, the AFB1 content in rice from the experimental warehouse was significantly lower than that in the control warehouse at equivalent timepoints and storage layers (*p* < 0.05). Integrated with baseline data from Table 2, the incremental increase in AFB1 in the experimental warehouse was markedly smaller than in the control warehouse. These results, combined with data from Section 3.6, demonstrate that integrated CO_2_-modified atmosphere and temperature-control technology effectively inhibits the proliferation of toxigenic fungi and curbs the accumulation rate of AFB1 in stored rice. Mannaa et al. (2018) [45] demonstrated that maintaining lower temperatures (10–20 °C) during rice storage suppresses the accumulation of AFB1, consistent with our experimental observations.

### 3.9. Metabolite Analysis of Rice Under Different Storage Conditions

To validate the impact of integrated CO_2_-modified atmosphere and temperature-control technology on rice metabolites in physical warehouses, metabolites in rice samples were profiled using UHPLC-OE-MS. The profiled metabolites encompassed amino acids, carbohydrates, fatty acids, and related compounds.

#### 3.9.1. Principal Component Analysis (PCA)

PCA is a dimensionality-reduction method that transforms potentially correlated variables into a set of linearly uncorrelated principal components through orthogonal transformation [20]. This process simplifies data structure and enhances computational efficiency. In the resulting score plot, the coordinates PC1 and PC2 represent the first and second principal components, respectively. Each scatter point denotes an individual sample, with distinct colors indicating different sample groups. The 95% confidence ellipse delineates the distribution boundary for each group. Unsupervised PCA of LC-MS-identified metabolites was employed to assess inter-group and intra-group variations.

**Figure 5:**
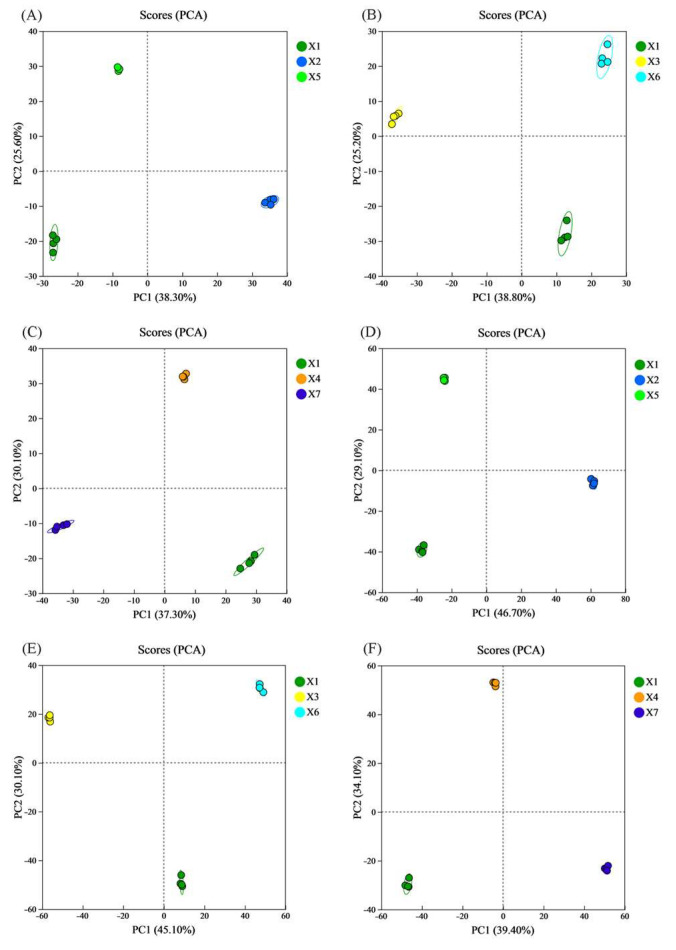
Changes in the PCA score plots of rice samples that were stored under various conditions. X1 corresponds to the origin; X2, X3, and X4 correspond to the top, middle, and bottom rice samples of the experimental warehouse; X5, X6, and X7 correspond to the top, middle, and bottom rice samples of the control warehouse. ((**A**–**C**) are the positive ion mode; (**D**–**F**) are in negative ion mode).

As shown in Figure 5, pronounced metabolic disparities were detected between the upper, middle, and lower layers of the experimental warehouse and their corresponding layers in the control warehouse after 360 days of storage, under both (A)–(C) positive ion and (D)–(F) negative ion modes. Significant differences were also observed between the experimental/control warehouses and the pre-storage origin rice. In contrast, intra-group metabolic variations remained negligible. As exemplified in Figure 5A, the PCA score plot under positive ion mode distinctly segregates rice samples from three groups—the upper layer of the experimental silo, the corresponding control silo layer, and pre-storage source rice—into three independent clusters based on principal component 1 (PC1) and principal component 2 (PC2). The first principal component (PC1) and the second principal component (PC2) collectively accounted for 64% of the total sample variance, with PC1 alone contributing 38.3%. A clear separation among the four experimental groups was observed along the PC1 dimension. All samples fell within the 95% confidence intervals, demonstrating that the PCA results were both reliable and reproducible. This result demonstrates that the characteristic patterns of significantly different metabolites between groups are influenced by storage time and storage conditions. A high degree of dispersion between groups, with no overlap, indicates substantial metabolic differences. In contrast, the limited dispersion among the four similarly colored points suggests minimal intra-group metabolic variation. Similar trends were observed in Figure 5B–F. These findings indicate that after 360 days of storage, the combined CO_2_-modified atmosphere and temperature-control technology significantly altered rice metabolism, resulting in marked differences compared to rice stored in conventional conditions at the same storage level.

#### 3.9.2. Orthogonal Projections to Latent Structures Discriminant Analysis (OPLS-DA)

As an unsupervised dimensionality reduction method, PCA may be confounded by extraneous variables unrelated to experimental grouping, potentially obscuring inter-group differences. To address this limitation, we introduce OPLS-DA—a supervised discriminant analysis method designed to enhance the resolution of inter-sample variations. OPLS-DA, a supervised discriminant analysis method, was employed to identify differential metabolites, predict sample categories, and visualize inter-group separation [46]. In the score plot, each scatter represents an individual sample. The confidence ellipse (95% probability density) delineates group boundaries, where a larger horizontal distance between group centroids indicates greater inter-group disparity, while a smaller vertical dispersion reflects higher intra-group homogeneity.

**Figure 6:**
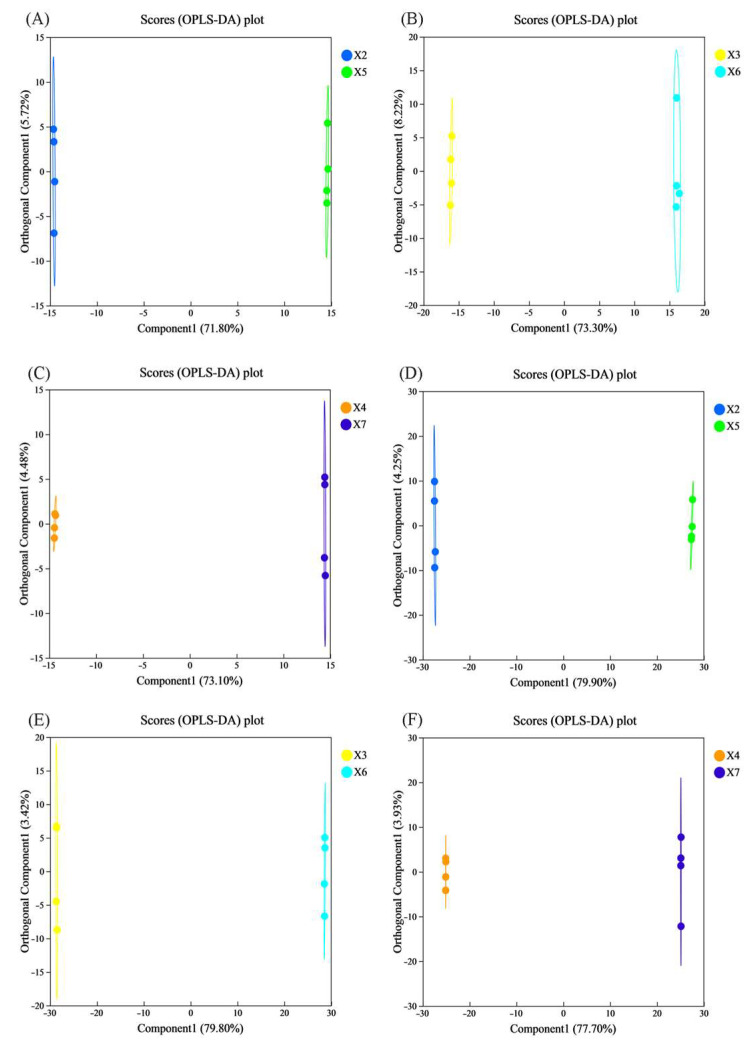
Changes in the OPLS-DA score plots of rice samples that were stored under various conditions. X2, X3, and X4 correspond to the top, middle, and bottom rice samples of the experimental warehouse; X5, X6, and X7 correspond to the top, middle, and bottom rice samples of the control warehouse. ((**A**–**C**) are the positive ion mode; (**D**–**F**) are in negative ion mode).

As illustrated in Figure 6A–C (positive ion mode) and Figure 6D–F (negative ion mode), significant metabolic differences were observed between the upper, middle, and lower layers of rice stored for 360 days in the experimental warehouse and their counterparts in the control warehouse. Taking Figure 6B as an exemplar (experimental vs. control silo, middle layer, positive ion mode), the OPLS-DA score plot displays a clear inter-group separation along predictive component 1 (73.3% explained variance), with the orthogonal component accounting for 8.22% of variance. The pronounced separation along the component 1-axis—without overlap in the 95% confidence ellipses—indicates substantial metabolic disparity between storage conditions within the same layer. Results from Figure 6A,C–F consistently aligned with Figure 6B, demonstrating that CO_2_-modified atmosphere storage combined with temperature regulation significantly altered the metabolic profiles of rice after 360 days of storage, showing substantial disparity from the control silo at the corresponding layers.

#### 3.9.3. Identification of Key Metabolites in Rice

OPLS-DA-derived VIP values (first predictive component) quantified metabolite contributions to group discrimination. Differential metabolites were screened by VIP > 1, *p* < 0.05 (*t*-test), and FC > 2, then visualized in volcano plots: red—significant upregulation (log2FC > 1, *p* < 0.05), blue—significant downregulation (log2FC < −1, *p* < 0.05), and gray—non-significant (*p* ≥ 0.05).

**Figure 7:**
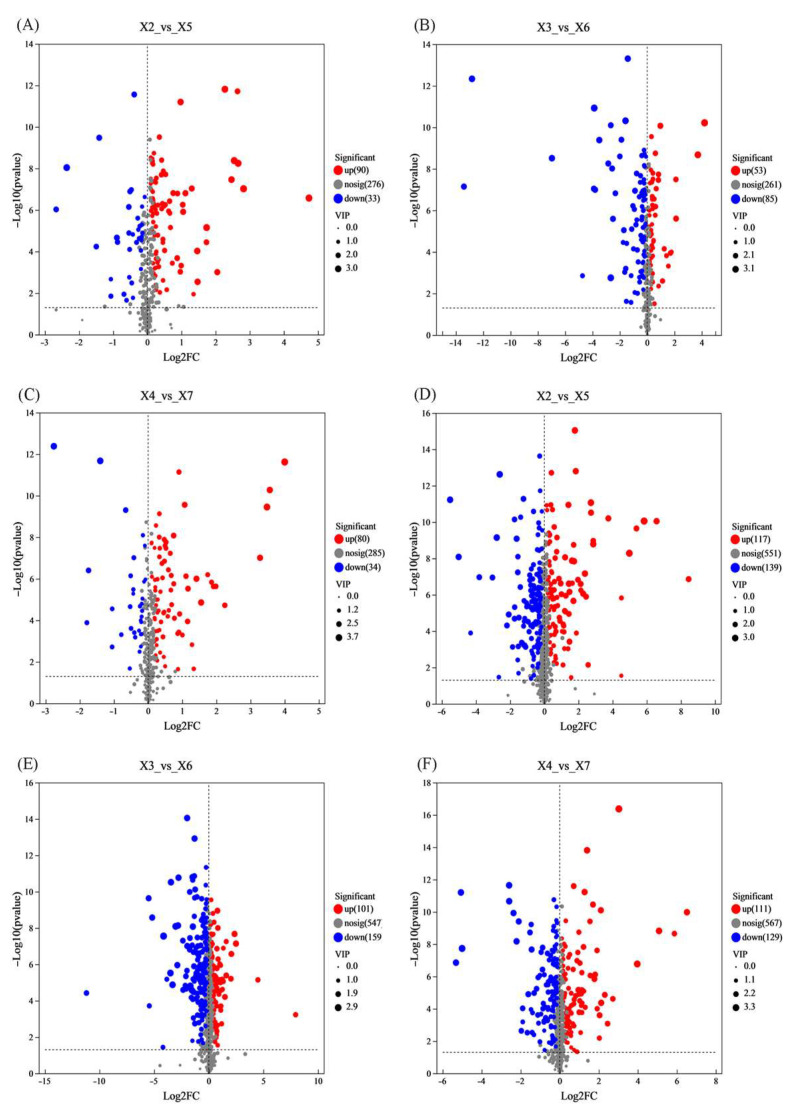
Changes in the volcano plots of rice samples that were stored under various conditions. X2, X3, and X4 correspond to the top, middle, and bottom rice samples of the experimental warehouse; X5, X6, and X7 correspond to the top, middle, and bottom rice samples of the control warehouse. ((**A**–**C**) are the positive ion mode; (**D**–**F**) are in negative ion mode).

In the same layer, rice in the experimental warehouse was compared with the control warehouse. The results of the positive ion mode analysis (Figure 7A–C) are as follows: upper layer—90 metabolites significantly upregulated, 33 downregulated; middle layer—53 metabolites upregulated, 85 downregulated; And lower layer—80 metabolites upregulated, 34 downregulated. The results of the negative ion mode analysis (Figure 7D–F) are as follows: upper layer—117 metabolites upregulated, 139 downregulated; middle layer—101 metabolites upregulated, 159 downregulated; and lower layer—111 metabolites upregulated, 129 downregulated.

#### 3.9.4. VIP Analysis of Differential Metabolites in Rice

**Figure 8:**
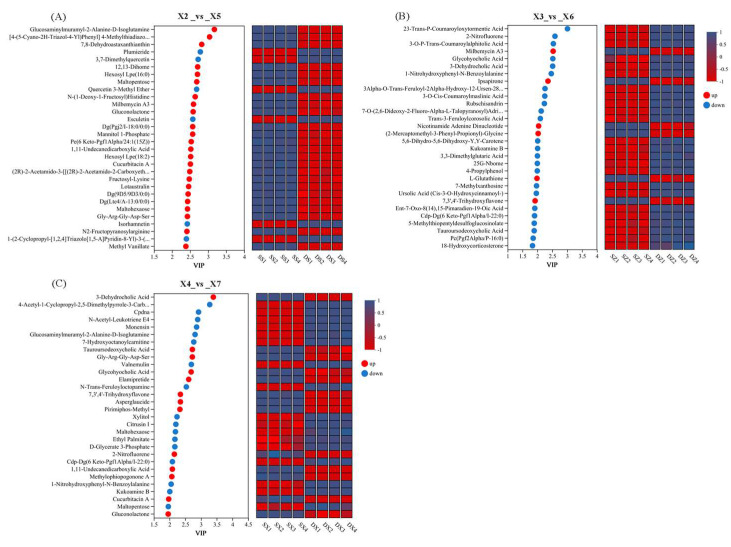
(**A**) Heat map of metabolite clustering in the X2 vs. X5 groups, (**B**) heat map of metabolite clustering in the X3 vs. X6 groups, and (**C**) heat map of metabolite clustering in the X4 vs. X7 groups. X2, X3, and X4 correspond to the top, middle, and bottom rice samples of the experimental warehouse; X5, X6, and X7 correspond to the top, middle, and bottom rice samples of the control warehouse.

After screening differential metabolites, VIP values were analyzed to identify the top 30 metabolites with the highest VIP values within each comparison group. Hierarchical clustering heat maps (Figure 8) visualized these metabolites across three spatial layers. Color gradients (red: upregulation; blue: downregulation) reflect log_2_-transformed abundance, enabling intuitive comparison of metabolite levels at each sampling point.

Within the same vertical layer, comparative analysis of differential metabolites revealed divergent profiles in rice subjected to different storage conditions. Upper layer (Figure 8A): Significant upregulation of Glucosaminylmuramyl-2-Alanine-D-Isoglutamine and [4-(5-Cyano-2H-Triazol-4-Yl) Phenyl] 4-Methylthiadiazole-5-Carboxylate, alongside 7, 8-Dehydroastaxanthianthin (carotenoid derivative). Conversely, Plumieride and 3,7-Dimethylquercetin were downregulated. Middle layer (Figure 8B): Downregulation dominated for stress-responsive metabolites including 23-Trans-P-Coumaroyloxytormentic Acid, 2-Nitrofluorene (xenobiotic), and 3-O-p-trans-coumaroylalphitolic acid. In contrast, Milbemycin A3 and Ipsapirone were upregulated. Lower layer (Figure 8C): Significant upregulation of bile acid derivatives (3-Dehydrocholic Acid, Tauroursodeoxycholic Acid) and the adhesion peptide Gly-Arg-Gly-Asp-Ser. Downregulation occurred for 4-Acetyl-1-Cyclopropyl-2,5-Dimethylpyrrole-3-Carboxylic Acid and chloroplast DNA (cpDNA, plant organelle genetic material).

#### 3.9.5. Functional Enrichment of KEGG Metabolic Pathways in Rice

To investigate the synergistic effects of CO_2_-modified atmosphere combined with temperature-modulated technology on metabolic processes in stored rice, differentially expressed metabolites from three spatial comparison groups—upper, middle, and lower layers (experimental warehouse vs. control warehouse)—were subjected to KEGG pathway enrichment analysis. Topological screening of enriched pathways identified representative key metabolic pathways for each comparison group. Results were visualized via KEGG topology maps, where each bubble denotes a KEGG pathway, with bubble size indicating pathway impact and color gradient reflecting −log (*p*-value).

**Figure 9:**
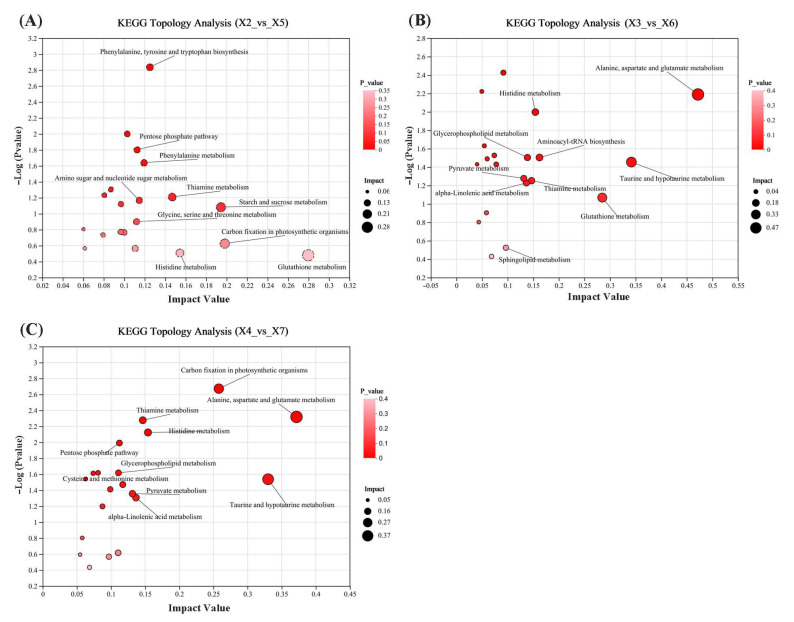
(**A**) X2 vs. X5 group KEGG topological analysis, (**B**) X3 vs. X6 group KEGG topological analysis, and (**C**) X4 vs. X7 group KEGG topological analysis. X2, X3, and X4 correspond to the top, middle, and bottom rice samples of the experimental warehouse; X5, X6, and X7 correspond to the top, middle, and bottom rice samples of the control warehouse.

Figure 9 shows the spatial stratification of key metabolic pathways in stored rice based on topological impact analysis. In the upper layer (Figure 9A), the top 10 pathways ranked by impact value are as follows: glutathione metabolism, carbon fixation in photosynthetic organisms, starch and sucrose metabolism, histidine metabolism, thiamine metabolism, phenylalanine, tyrosine and tryptophan biosynthesis, phenylalanine metabolism, amino sugar and nucleotide sugar metabolism, pentose phosphate pathway, and glycine, serine, and threonine metabolism. In the middle layer (Figure 9B), alanine, aspartate, and glutamate metabolism, taurine and hypotaurine metabolism, glutathione metabolism, aminoacyl-tRNA biosynthesis, histidine metabolism, thiamine metabolism, glycerophospholipid metabolism, pyruvate metabolism, alpha-linolenic acid metabolism, alpha-linolenic acid metabolism, and sphingolipid metabolism. In the lower layer (Figure 9C), alanine, aspartate and glutamate metabolism, taurine and hypotaurine metabolism, carbon fixation in photosynthetic organisms, alpha-linolenic acid metabolism, pyruvate metabolism, histidine metabolism, thiamine metabolism, pentose phosphate pathway, glycerophospholipid metabolism, and cysteine and methionine metabolism.

In summary, the recurrently enriched metabolic pathways demonstrated significant convergence with core biomolecular networks, particularly amino acid homeostasis (alanine, aspartate, and glutamate metabolism), lipid remodeling (alpha-linolenic acid metabolism), carbohydrate flux (pentose phosphate pathway), and vitamin-dependent cofactor regulation (thiamine metabolism).

### 3.10. Analysis of Metabolic Pathways

#### 3.10.1. Amino Acid Metabolism

**Figure 10:**
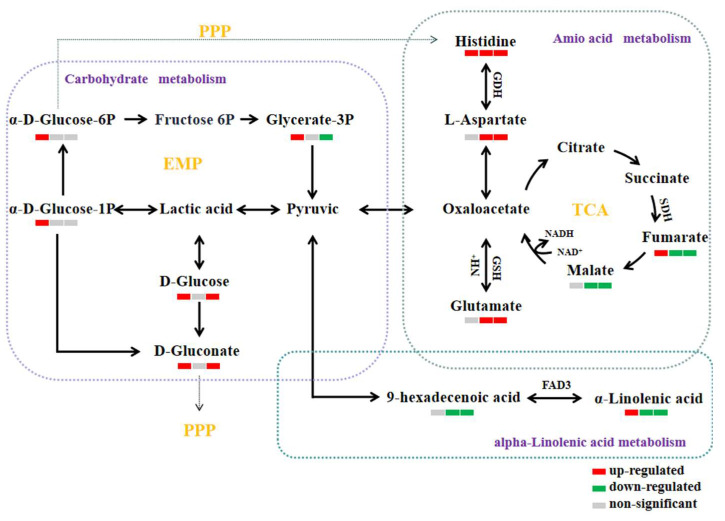
Relation of metabolites in rice under different conditions. Colors indicate metabolites that are upregulated (red) and downregulated (green), and those with no significant changes (gray). The figure shows a comparison of the different storage conditions, from left to right as follows: X2 vs. X5, X3 vs. X6, and X4 vs. X7. (X2, X3, and X4 correspond to the top, middle, and bottom rice samples of the experimental warehouse; X5, X6, and X7 correspond to the top, middle, and bottom rice samples of the control warehouse.)

Amino acids serve as central metabolic regulators enabling plants to adapt to environmental stresses and participate in diverse specialized metabolic processes. As primary metabolic intermediates, amino acids contribute to reactive oxygen species (ROS) scavenging through both direct antioxidant activity (e.g., metal chelation) and indirect redox signaling pathways [47]. As shown in Figure 10, pathway analysis revealed that differentially accumulated amino acids predominantly converged in the alanine–aspartate–glutamate metabolic axis and histidine biosynthesis and catabolism pathways. L-aspartate, glutamate, and histidine function as core metabolic hubs. These amino acids interconvert via the tricarboxylic acid cycle (TCA cycle) and glutamine synthetase-mediated reactions, forming an integrated metabolic network that coordinates carbon flux and nitrogen homeostasis.

In this experiment, compared to the middle and lower layers of the control warehouse, the contents of fumaric acid and malic acid (key TCA cycle intermediates) in the experimental warehouse were significantly decreased, while L-aspartate, glutamate, and histidine levels were markedly elevated. These data suggest that CO_2_-atmosphere regulation combined with temperature-control technology promotes the conversion of TCA cycle intermediates (e.g., fumarate and malate) into oxaloacetate, thereby channeling carbon flux toward the L-aspartate–histidine–glutamate metabolic axis. This shift enhances ROS scavenging capacity, collectively bolstering the antioxidant potential of stored rice.

L-aspartate functions as a pivotal metabolic intermediate in the aforementioned amino acid network and plays a critical role in maintaining metabolic homeostasis. For instance, L-aspartate participates in nitrogen recycling and is converted to histidine via glutamate dehydrogenase (GDH) catalysis. This process stabilizes the tertiary structure of storage proteins in rice grains during storage, thereby delaying protein degradation kinetics [48]. Additionally, L-aspartate exhibits direct antioxidant capacity by scavenging free radicals, protecting rice from oxidative damage [49]. Therefore, CO_2_-modified atmosphere regulation combined with temperature-control technology delays protein degradation and enhances free radical scavenging in grain cells during storage. This synergy improves rice storage stability and antioxidant properties, ultimately postponing the biochemical deterioration of stored rice quality.

Glutamate functions as a pivotal nitrogen metabolism hub and serves as the primary precursor for glutathione (GSH) synthesis. Its upregulated levels enhance cellular antioxidant capacity by supplying GSH, which suppresses reactive oxygen species (ROS) accumulation, thereby improving cellular oxidative defense and stabilizing membrane integrity [50]. This mechanism protects cells from lipid peroxidation-induced damage [51], contributing to the maintenance of rice quality stability during storage.

Histidine serves as a critical enzymatic cofactor beyond its fundamental role in protein architecture, and functions as a conditional essential amino acid in cereal storage systems. Through secondary metabolite biosynthesis pathways, histidine acts as a precursor for key biomolecules (e.g., histamine and carnosine) that regulate redox homeostasis and pathogen defense [52,53]. CO_2_-modified atmosphere regulation combined with temperature-control technology synergistically stabilizes histidine-dependent metabolic flux, thereby enhancing antioxidant capacity and disease resistance during storage. This integrated approach maintains rice freshness by preserving metabolic equilibrium and suppressing quality deterioration.

#### 3.10.2. Carbohydrate Metabolism

As shown in Figure 10, the pentose phosphate pathway (PPP) constitutes a fundamental glucose-metabolizing axis alongside glycolysis and the tricarboxylic acid (TCA) cycle, collectively forming the core respiratory metabolic network in cells [54]. The pentose phosphate pathway constitutes a pivotal glucose-metabolizing pathway in organisms. Its primary functions encompass the following: generating nicotinamide adenine dinucleotide phosphate (NADPH), PPP for nucleic acid metabolism, and producing key intermediates involved in amino acid and fatty acid synthesis [55].

Gluconic acid and gluconolactone are pivotal intermediates in the pentose phosphate pathway, synthesized via secondary metabolite formation or carbon metabolism [56]. Studies indicate that both compounds potentiate cellular antioxidant defense by scavenging reactive oxygen species (ROS) and activating enzymatic antioxidants (e.g., superoxide dismutase/SOD, catalase/CAT), thereby mitigating oxidative damage in plant cells [57]. In this experiment, the contents of gluconic acid and gluconolactone were significantly elevated in both the upper and lower layers of the experimental warehouse compared to the control. This indicates that CO_2_-modified atmosphere technology combined with temperature modulation may attenuate oxidative damage in rice cells by maintaining redox homeostasis of grain cells, thereby delaying grain senescence and enhancing storage stability.

#### 3.10.3. Lipid Metabolism

As shown in Figure 10, α-linolenic acid (ALA) is an essential polyunsaturated fatty acid of the n-3 series, exhibiting cardioprotective, anti-inflammatory, and neuroprotective properties in human health. However, rice grains contain suboptimal concentrations of ALA, which is vulnerable to oxidative degradation during storage, potentially compromising nutritional stability. Notably, palmitoleic acid (9-hexadecenoic acid) is a key Δ6-desaturation metabolite of ALA, synthesized via FADS2-catalyzed enzymatic conversion [58]. Heilborn et al. (2023) [59] demonstrated that elevated oxygen concentrations accelerate the metabolic flux from ALA to palmitoleic acid, indicating oxygen-dependent regulation of this desaturation pathway. In this experiment, the palmitoleic acid content in rice stored in the middle and lower layers of the experimental warehouse was significantly reduced compared to the control warehouse. This indicates that CO_2_-modified atmosphere technology attenuated the metabolic conversion of ALA to palmitoleic acid. Mechanistically, elevated CO_2_ concentrations lowered oxygen availability within the grain pile, thereby suppressing FADS2-mediated Δ6-desaturation of ALA [60]. Consequently, this intervention delayed oxidative degradation of functional lipids and better preserved the nutritional value of rice during storage.

## 4. Conclusions

This study innovatively integrated CO_2_-modified atmosphere and temperature-controlled technology, systematically validating for the first time the “physical-biochemical” dual-path mechanism regulating rice grain quality in an actual warehouse storage environment. Through non-targeted metabolomics, differential metabolites were precisely identified, revealing that the integrated technology synergistically modulates amino acid, carbohydrate, and lipid metabolism in rice. Experiments confirmed that during the 360-day storage period, the use of CO_2_-modified atmosphere combined with temperature-control technology significantly slowed the progression of changes in rice color, FAV, brown rice yield, head milled rice yield, and germination percentage. This approach effectively suppressed the metabolic activity of harmful molds and reduced the extent of increase in AFB1 content. Furthermore, by modulating the metabolism of amino acids, carbohydrates, and lipids, the oxidative stress resistance of rice was significantly enhanced, leading to better retention of nutritional components such as amino acids and proteins. In summary, this technology has delayed the quality degradation of rice, suppressed biological contamination, and enhanced its stress resistance. The low cost and environmentally friendly nature of carbon dioxide employed in this technology offer a new approach to reducing the use of chemical agents in practical warehouse storage, lowering economic costs, and promoting the transformation of the grain storage industry from a “high-energy fumigation” model to “low-carbon intelligent storage”.

## Figures and Tables

**Table 1:** Sampling metadata for fungal diversity analysis in stored rice.

Sample Information	Sample Name	Sample Grouping
Origin	AB	AB
Experimental warehouse, upper	A1	A
Experimental warehouse, middle	A2	A
Experimental warehouse, lower	A3	A
Control warehouse, upper	B1	B
Control warehouse, middle	B2	B
Control warehouse, lower	B3	B

**Table 2:** Origin quality parameters of stored rice.

Quality Indicators	Content	Units
L*	58.80 ± 0.17	-
a*	4.27 ± 0.58	-
b*	15.53 ± 0.58	-
Fatty acid value	14.23 ± 0.07	(mg/100 g)
Brown rice yield	78.93 ± 0.21	(%)
Head milled rice yield	62.13 ± 0.17	(%)
Germination percentage	90.33 ± 0.17	(%)
Aflatoxin B_1_	1.66 ± 0.02	(μg/kg)

## Data Availability

The original contributions presented in the study are included within the article; further inquiries can be directed to the corresponding author.

## References

[1] Martens S., Coradi P.C., Maldaner V., de Oliveira Carneiro L., Teodoro P.E., Rodrigues D.M., Anschau K.F., Teodoro L.P.R., Flores É.M.M. (2023). Drying and intermittence processes on the polished and brown rice physicochemical and morphological quality by near-infrared spectroscopy, X-ray diffraction, and scanning electron microscopy. Food Chem. X.

[2] Zhao Q., Guo H., Hou D., Laraib Y., Xue Y., Shen Q. (2021). Influence of temperature on storage characteristics of different rice varieties. Cereal Chem..

[3] Zhao Y., Li Y., Gong Z., Liu X., Lv H., Zhao Y. (2024). Changes in Quality Characteristics and Metabolite Composition of LowTemperature and Nitrogen-Modified Atmosphere in Indica Rice during Storage. Foods.

[4] Perišić V., Perišić V., Hadnađev M., Đekić V., Dapčević-Hadnađev T., Vuković S., Vukajlović F. (2019). Impact of diatomaceous earth application on the rheological properties of wheat, triticale and rye flour dough. J. Stored Prod. Res..

[5] Chen W., Xu H., Chen M., Tang P., Wang K. (2025). Spray-Induced Gene Silencing for Postharvest Protection: dsRNA Stability and Insecticidal Efficacy. J. Agric. Food Chem..

[6] Moirangthem T.T., Baik O.-D. (2021). Disinfestation of stored grains using non-chemical technologies—A review. Trends Food Sci. Technol..

[7] Lin J., Yan X., Zhou Y., Zhu G., Ariyo O.S., Wang S., Chen J., Zhu Y., Wang C., Li D. (2025). Modified atmosphere and ozone treatment technologies in stored grain pest control: Mechanism, applications and challenges. Agric. Prod. Process. Storage.

[8] Navarro S. (2012). The use of modified and controlled atmospheres for the disinfestation of stored products. J. Pest Sci..

[9] Anuja Gupta A.G., Sinha S.N., Atwal S.S. (2014). Modified atmosphere technology in seed health management: Laboratory and field assay of carbon dioxide against storage fungi in paddy. Plant Pathol. J..

[10] Sun S., Li B., Yang T., Luo F., Zhao J., Cao J., Lin Q. (2019). Preservation mechanism of high concentration carbon dioxide controlled atmosphere for paddy rice storage based on quality analyses and molecular modeling tools. J. Cereal Sci..

[11] Xue G., Wang T., Guo H., Zhang N., Carmalt C.J., Hofkens J., Lai F., Liu T. (2025). Polymer-confined synthesis of gram-scale high-entropy perovskite fluoride nanocubes for improved electrocatalytic reduction of nitrate to ammonia. Nanoscale Horiz..

[12] Hamzavi F., Naseri B., Hassanpour M., Razmjou J., Golizadeh A. (2022). Biology and life table parameters of *Callosobruchus maculatus* (F.) on *Vigna unguiculata* (L.) Walp. fertilized with some mineral-and bio-fertilizers. J. Stored Prod. Res..

[13] Han Q., Chen Y., Liu X., Bi J., Zhang W., Zeng X., Wang P., Shu Z. (2024). Quality attributes of paddy rice during storage as affected by accumulated temperature. Front. Nutr..

[14] Qu L., Zhao Y., Xu X., Li Y., Lv H. (2023). Untargeted Lipidomics Reveal Quality Changes in High-Moisture Japonica Brown Rice at Different Storage Temperatures. Foods.

[15] Abdelfattah A., Whitehead S.R., Macarisin D., Liu J., Burchard E., Freilich S., Dardick C., Droby S., Wisniewski M. (2020). Effect of Washing, Waxing and Low-Temperature Storage on the Postharvest Microbiome of Apple. Microorganisms.

[16] Hu X.Q., Lu L., Guo Z.L., Zhu Z.W. (2020). Volatile compounds, affecting factors and evaluation methods for rice aroma: A review. Trends Food Sci. Technol..

[17] Chen L., Lu W., Wang L., Xing X., Chen Z., Teng X., Zeng X., Muscarella A.D., Shen Y., Cowan C.A. (2021). Metabolite discovery through global annotation of untargeted metabolomics data. Nat. Methods.

[18] Chen C., Qiao X., Guo J., Yang T., Wang M., Ma Y., Zhao S., Ding L., Liu H., Wang J. (2022). Related factors based on non-targeted metabolomics methods in minor ischaemic stroke. Front. Endocrinol..

[19] Xu J.Y., Chen Z., Wang F.Z., Jia W., Xu Z.C. (2020). Combined transcriptomic and metabolomic analyses uncover rearranged gene expression and metabolite metabolism in tobacco during cold acclimation. Sci. Rep..

[20] Li W., Zhou Y., Zhang H., Hu M., Lu P., Qu C. (2024). Study on peanut protein oxidation and metabolomics/proteomics analysis of peanut response under hypoxic/re-aeration storage. Food Chem. X.

[21] Wu Y.N., He S.D., Pan T.E., Miao X.Y., Xiang J., Ye Y.K., Cao X.D., Sun H.J. (2023). Enhancement of gamma-aminobutyric acid and relevant metabolites in brown glutinous rice (*Oryza sativa* L.) through salt stress and low-frequency ultrasound treatments at pre-germination stage. Food Chem..

[22] Zhang Y., He C., Wu Y., Yang J., Xuan H., Zhu X. (2009). Effect of lipoxygenase activity and red seed coat on rice bran deterioration. J. Sci. Food Agric..

[23] Dou Z., Zhou Y.C., Zhang Y.Y., Guo W., Xu Q., Gao H. (2024). Influence of nitrogen applications during grain-filling stage on rice (*Oryza sativa* L.) yield and grain quality under high temperature. Agronomy.

[24] Shad Z.M., Atungulu G. (2020). Physical integrity of long-Grain hybrid, pureline, and medium-grain rice kernels as affected by storage conditions. Appl. Eng. Agric..

[25] Chao S., Mitchell J., Fukai S. (2021). Factors determining genotypic variation in the speed of rice germination. Agronomy.

[26] Alwan N., Bou Ghanem H., Dimassi H., Karam L., Hassan H.F. (2022). Exposure assessment of aflatoxin B1 through consumption of rice in the United Arab Emirates. Int. J. Environ. Res. Public Health.

[27] Xiao W., Ding Y., Cheng Y., Xu S., Lin L. (2022). Understanding the Changes in Quality of Semi-Dried Rice Noodles during Storage at Room Temperature. Foods.

[28] Kibar H., Sönmez F., Temel S. (2021). Effect of storage conditions on nutritional quality and color characteristics of quinoa varieties. J. Stored Prod. Res..

[29] Gao B., Hu S., Jing L., Wang Y., Zhu J., Wang K., Li H., Sun X., Wang Y., Yang L. (2021). Impact of elevated CO_2_ and reducing the source-sink ratio by partial defoliation on rice grain quality-A 3-year free-air CO_2_ enrichment study. Front. Plant Sci..

[30] Genkawa T., Uchino T., Inoue A., Tanaka F., Hamanaka D. (2008). Development of a low-moisture-content storage system for brown rice: Storability at decreased moisture contents. Biosyst. Eng..

[31] Wang T., She N., Wang M., Zhang B., Qin J., Dong J., Fang G., Wang S. (2021). Changes in Physicochemical Properties and Qualities of Red Brown Rice at Different Storage Temperatures. Foods.

[32] Tian J., Ji G., Zhang J., Luo D., Zhang F., Li L., Jiang M., Zhu D., Li M. (2025). Evaluation of Rice Quality Storage Stability: From Variety Screening to Trait Identification. Plants.

[33] Ziegler V., da Silveira E.B., Dalla Vecchia V., Ferreira C.D. (2024). Chilled paddy rice grains applied directly to industrial processing have a better head rice yield. J. Stored Prod. Res..

[34] Wang X., Pang Y., Wang C., Chen K., Zhu Y., Shen C., Ali J., Xu J., Li Z. (2017). New candidate genes affecting rice grain appearance and milling quality detected by genome-wide and gene-based association analyses. Front. Plant Sci..

[35] Liu S., Waqas M.A., Wang S.H., Xiong X.Y., Wan Y.F. (2017). Effects of increased levels of atmospheric CO_2_ and high temperatures on rice growth and quality. PLoS ONE.

[36] Hu H., Li S., Pan D., Wang K., Qiu M., Qiu Z., Liu X., Zhang J. (2022). The variation of rice quality and relevant starch structure during long-term storage. Agriculture.

[37] Aguiar R.D.S., Brito D.R., Lopes M.D.M., Silva R.R., Fidelis R.R., Sousa C.D., Santos G.D. (2015). Effect of carbon dioxide on quality of rice seeds. Biosci. J..

[38] Hashem A.S., Guedes R.N.C., Awadalla H.S. (2021). Feeding substrate and temperature interplay determining infestations and losses by the sawtoothed grain beetle (*Oryzaephilus surinamensis*). J. Stored Prod. Res..

[39] Gomdola D., McKenzie E.H., Bundhun D., Jayawardena R.S. (2024). Morpho-molecular characterization of phoma-like fungi from Morus alba in northern Thailand; a novel species (*Boeremia albae*) and a new host record (*B. maritima*). Fungal Biol..

[40] Femenias A., Gatius F., Ramos A.J., Teixido-Orries I., Marín S. (2022). Hyperspectral imaging for the classification of individual cereal kernels according to fungal and mycotoxins contamination: A review. Food Res. Int..

[41] Kushwaha R., Singh V., Kaur S., Kaur D. (2023). Modulating the characteristics of jackfruit seed starches by annealing and autoclaving-cooling modifications. J. Food Process Eng..

[42] Geng Q., Hu J., Xu P., Sun T., Qiu H., Wang S., Song F., Shen L., Li Y., Liu M. (2024). The Autophagy-Related Protein ATG8 Orchestrates Asexual Development and AFB1 Biosynthesis in *Aspergillus flavus*. J. Fungi.

[43] Amaike S., Keller N.P. (2011). *Aspergillus* *flavus*. Annu. Rev. Phytopathol..

[44] Ouma F., Luthra K., Oduola A., Atungulu G.G. (2024). Investigating safe storage conditions to mitigate aflatoxin contamination in rice. Food Control.

[45] Mannaa M., Kim K.D. (2018). Effect of temperature and relative humidity on growth of *Aspergillus* and *Penicillium* spp. and biocontrol activity of *Pseudomonas protegens* AS15 against aflatoxigenic *Aspergillus flavus* in stored rice grains. Mycobiology.

[46] Yates A.G., Dierksmeier S., Couch Y., Claridge T.D., Probert F., Anthony D.C., Ruitenberg M.J. (2025). Lesion level and severity acutely influence metabolomic profiles in spinal cord injury. J. Neuropathol. Exp. Neurol..

[47] Kadam S.B., Barvkar V.T. (2024). COI1 dependent jasmonic acid signalling positively modulates ROS scavenging system in transgenic hairy root culture of tomato. Plant Physiol. Biochem..

[48] Li Z., Bhowmik S., Sagresti L., Brancato G., Smith M., Benson D.E., Li P., Merz K.M. (2024). Simulating metal-imidazole complexes. J. Chem. Theory Comput..

[49] Hu Y., Liu Y., Cheng C. (2013). Production, application and market prospect of L-aspartic acid. Food Ferment. Ind..

[50] Xu L., Song J.Q., Wang Y.L., Liu X.H., Li X.L., Zhang B., Li A.J., Ye X.F., Wang J., Wang P. (2022). Thymol improves salinity tolerance of tobacco by increasing the sodium ion efflux and enhancing the content of nitric oxide and glutathione. BMC Plant Biol..

[51] Syeed S., Sehar Z., Masood A., Anjum N.A., Khan N.A. (2021). Control of elevated ion accumulation, oxidative stress, and lipid peroxidation with salicylic acid-induced accumulation of glycine betaine in salinity-exposed *Vigna radiata* L.. Appl. Biochem. Biotechnol..

[52] Holeček M. (2020). Histidine in health and disease: Metabolism, physiological importance, and use as a supplement. Nutrients.

[53] Thalacker-Mercer A.E., Gheller M.E. (2020). Benefits and adverse effects of histidine supplementation. J. Nutr..

[54] Heidarvand L., Millar A.H., Taylor N.L. (2017). Responses of the mitochondrial respiratory system to low temperature in plants. Crit. Rev. Plant Sci..

[55] Xu Y., Schmiege S.C., Sharkey T.D. (2024). The oxidative pentose phosphate pathway in photosynthesis: A tale of two shunts. New Phytol..

[56] Phégnon L., Pérochon J., Uttenweiler-Joseph S., Cahoreau E., Millard P., Létisse F. (2024). 6-Phosphogluconolactonase is critical for the efficient functioning of the Pentose phosphate pathway. FEBS J..

[57] Zhang F., Liu Q., Wang Y., Yin J., Meng X., Wang J., Zhao W., Liu H., Zhang L. (2024). Effects of Surfactin Stress on Gene Expression and Pathological Changes in Spodoptera Litura. Sci. Rep..

[58] Ge C., Chen H., Mei T., Tang X., Chang L., Gu Z., Zhang H., Chen W., Chen Y.Q. (2018). Application of a ω-3 desaturase with an arachidonic acid preference to eicosapentaenoic acid production in *Mortierella alpina*. Front. Bioeng. Biotechnol..

[59] Heidler von Heilborn D., Reinmüller J., Yurkov A., Stehle P., Moeller R., Lipski A. (2023). Fungi under Modified Atmosphere—The Effects of CO_2_ Stress on Cell Membranes and Description of New Yeast *Stenotrophomyces fumitolerans* gen. nov., sp. nov. J. Fungi.

[60] Chorner Z., Barbeau P.A., Castellani L., Wright D.C., Chabowski A., Holloway G.P. (2016). Dietary α-linolenic acid supplementation alters skeletal muscle plasma membrane lipid composition, sarcolemmal FAT/CD36 abundance, and palmitate transport rates. Am. J. Physiol. Regul. Integr. Comp. Physiol..

